# Fracture Risk Among Living Kidney Donors 25 Years After Donation

**DOI:** 10.1001/jamanetworkopen.2023.53005

**Published:** 2024-01-24

**Authors:** Hilal Maradit Kremers, Brandon R. Grossardt, Adam R. Miller, Bertram L. Kasiske, Arthur J. Matas, Sundeep Khosla, Walter K. Kremers, Hatem Amer, Rajiv Kumar

**Affiliations:** 1 Division of Epidemiology, Department of Quantitative Health Sciences, Mayo Clinic, Rochester, Minnesota; 2 Department of Orthopedics, Mayo Clinic, Rochester, Minnesota; 3 Division of Clinical Trials and Biostatistics, Department of Quantitative Health Sciences, Mayo Clinic, Rochester, Minnesota; 4 William J. von Liebig Center for Transplantation and Clinical Regeneration, Mayo Clinic, Rochester, Minnesota; 5 Division of Nephrology, Department of Medicine, Hennepin County Medical Center, Minneapolis, Minnesota; 6 Division of Transplantation, Department of Surgery, University of Minnesota, Minneapolis; 7 Division of Endocrinology, Diabetes and Metabolism, Department of Medicine and Kogod Center on Aging, Mayo Clinic, Rochester, Minnesota; 8 Department of Physiology and Biomedical Engineering, Mayo Clinic, Rochester, Minnesota; 9 Division of Nephrology and Hypertension, Nephrology Research Unit, Department of Medicine, Mayo Clinic, Rochester, Minnesota; 10 Department of Biochemistry and Molecular Biology, Mayo Clinic, Rochester, Minnesota

## Abstract

**Importance:**

Living kidney donors may have an increased risk of fractures due to reductions in kidney mass, lower concentrations of serum 1,25-dihydroxyvitamin D, and secondary increases in serum parathyroid hormone.

**Objective:**

To compare the overall and site-specific risk of fractures among living kidney donors with strictly matched controls from the general population who would have been eligible to donate a kidney but did not do so.

**Design, Setting, and Participants:**

This survey study was conducted between December 1, 2021, and July 31, 2023. A total of 5065 living kidney donors from 3 large transplant centers in Minnesota were invited to complete a survey about their bone health and history of fractures, and 16 156 population-based nondonor controls without a history of comorbidities that would have precluded kidney donation were identified from the Rochester Epidemiology Project and completed the same survey. A total of 2132 living kidney donors and 2014 nondonor controls responded to the survey. Statistical analyses were performed from May to August 2023.

**Exposure:**

Living kidney donation.

**Main Outcomes and Measures:**

The rates of overall and site-specific fractures were compared between living kidney donors and controls using standardized incidence ratios (SIRs).

**Results:**

At the time of survey, the 2132 living kidney donors had a mean (SD) age of 67.1 (8.9) years and included 1245 women (58.4%), and the 2014 controls had a mean (SD) age of 68.6 (7.9) years and included 1140 women (56.6%). The mean (SD) time between donation or index date and survey date was 24.2 (10.4) years for donors and 27.6 (10.7) years for controls. The overall rate of fractures among living kidney donors was significantly lower than among controls (SIR, 0.89; 95% CI, 0.81-0.97). However, there were significantly more vertebral fractures among living kidney donors than among controls (SIR, 1.42; 95% CI, 1.05-1.83).

**Conclusions and Relevance:**

This survey study found a reduced rate of overall fractures but an excess of vertebral fractures among living kidney donors compared with controls after a mean follow-up of 25 years. Treatment of excess vertebral fractures with dietary supplements such as vitamin D_3_ may reduce the numbers of vertebral fractures and patient morbidity.

## Key Points

**Question:**

What is the long-term risk of overall and site-specific fractures among living kidney donors?

**Findings:**

In this survey study that included 2132 living kidney donors and 2014 controls, the overall rate of fractures was significantly lower among donors than controls. However, there were significantly more vertebral fractures among donors than controls.

**Meaning:**

This study suggests that, although the overall fracture rate among donors was low, reductions in kidney mass and prolonged hyperparathyroidism may predispose living kidney donors to trabecular bone loss and vertebral fractures.

## Teaser

This survey study compares the overall and site-specific risk of fractures among living kidney donors with strictly matched controls from the general population who would have been eligible to donate a kidney but did not do so.

## Introduction

The prevalence of living kidney donation is increasing. Each year, approximately 30 000 persons worldwide become living kidney donors.^1^ Since 1988 in the US, 187 184 persons have donated a kidney from among 459 849 transplants performed. In 2022 alone, from 20 093 kidney transplants performed in the US, 5865 (29.2%) were living donor transplants.^2^ Living kidney donation is not without health risks to the donor. For example, the 15-year observed risk of end-stage kidney disease (ESKD) in kidney donors, although low, is reported to be 3.5 to 5.3 times higher than the projected risk in the absence of donation.^3,4,5^ Studies to date have focused largely on the risk of ESKD after kidney donation, and few studies have examined other potential health consequences that are important to donors and their families. In an Australian study, 123 donors prioritized the importance of a list of 35 postdonation outcomes.^6^ Kidney function was the highest-ranked outcome, whereas bone health was ranked much lower.

The risk of bone disease and fractures is increased in chronic kidney disease (CKD) and ESKD. The mechanisms associated with the development of kidney osteodystrophy in the context of a reduction in glomerular filtration rate (GFR), including inorganic phosphate retention, reduced serum 1,25(OH)_2_D production, and secondary hyperparathyroidism, have been extensively studied.^7,8,9,10,11,12,13,14,15^ Bone quality is abnormal in CKD and is associated with a higher prevalence of hip and vertebral fractures among persons with ESKD compared with the general population among all age groups.^16,17^ Hip fractures among persons with ESKD are associated with a doubling of mortality compared with hip fractures among patients not undergoing dialysis.^18,19^

A prospective study previously measured markers of mineral and bone metabolism among 182 kidney donors after kidney donation and 173 paired nondonor controls.^20^ Donors had significantly higher serum intact parathyroid hormone and fibroblast growth factor 23 concentrations, significantly lower serum 1,25(OH)_2_D concentrations and measured GFR, and a reduced tubular reabsorption of inorganic phosphate 6 and 36 months after donation compared with healthy controls. Higher concentrations of bone resorption markers carboxyterminal cross-linking telopeptide of collagen, aminoterminal cross-linking telopeptide of collagen, and tartrate-resistant acid phosphatase 5b and of the bone formation markers procollagen type I N-terminal propeptide, bone alkaline phosphatase, and osteocalcin were observed among donors compared with healthy controls. Small retrospective or prospective studies examining a limited number of hormonal and bone turnover variables have shown similar findings.^21,22,23,24,25,26^ In these studies, bone formation was increased due to the well-recognized “coupling” of bone formation to bone resorption^27^; however, among adults, increased bone turnover is uniformly associated with an increase in bone resorption relative to formation, bone microarchitectural deterioration, and bone loss.^28^ Collectively, these studies suggest that bone quality might be impaired in kidney donors. Prolonged hyperparathyroidism is expected to predispose donors to trabecular bone loss and an increase in cortical bone, leading to an increase in axial skeleton fractures without any changes in appendicular skeletal fractures. To our knowledge, there are no studies to date with sufficiently long follow-up examining the risk of fractures among kidney donors. A Canadian study failed to demonstrate an excess risk of fractures among kidney donors, but the cohort included younger donors (mean age, 43 years) at low risk who had donated a mean of 6 years before participation in the study.^29^ Only 19% of the donors had a donor nephrectomy more than 12 years before the study. We posit that this study failed to observe an increased risk of fractures among donors because the excess risk of fractures would not be expected to occur until several years after donation and typically at 50 years of age or older. With this background, the goal of our study was to compare the long-term risk of fractures among living kidney donors with strictly matched controls from the general population.

## Methods

### Recruitment of Donors and Controls

The study was approved by the institutional review boards at Hennepin County Medical Center, the University of Minnesota Medical Center, and the Mayo Clinic, Rochester. The institutional review board of Mayo Clinic, Rochester served as the institutional review board of record for data used from controls. We identified 5065 persons who met the following criteria: (1) they had donated a kidney at Mayo Clinic, Rochester; Hennepin County Medical Center; or the University of Minnesota Medical Center; (2) they were at least 50 years of age (as of January 1, 2022); and (3) 10 years or more had lapsed after the date of kidney donation. Our study was focused on surveying these donors because they had been exposed to an altered hormonal milieu for at least 10 years,^20^ and they represented a population for whom the risk of fractures was increased because they had attained an age of at least 50 years.^30^ All survey participants signed the Authorization to Use and Disclose Protected Health Information (Health Insurance Portability and Accountability Act) form and returned it with the survey questionnaire, indicating their informed consent. This study followed the American Association for Public Opinion Research (AAPOR) reporting guideline.^31^

Using the population-based resources of the Rochester Epidemiology Project (REP), we also identified a strictly matched group of nondonor individuals who would have been eligible to donate but did not donate a kidney.^32^ The matched controls were identified within the REP population of persons who had at least 5 years of medical history prior to their age at matching (year of donation) to allow sufficiently long medical history information to assess their health status. Potential matches were added to a subset to include only those who were known to be alive. Matching criteria were age (±5 years), year of donation (index date), sex, and self-reported race and ethnicity. Race and ethnicity (racial or ethnic minority group, White, and unknown) for matching were available from the respective donor registries and the REP medical records linkage system. Racial or ethnic minority group was used as a category because we did not have access to the precise data in terms of breakdown of race and ethnicity, but donor and control groups were balanced. Race and ethnicity were assessed because of the association of race with bone density. Although age within 5 years was a requirement for matching, preference was given to nondonor individuals with a closer matching age. The medical records of all matched controls were electronically reviewed to exclude those with a history of comorbidities that would have precluded kidney donation, including heart disease, pulmonary disease, diabetes, liver disease, kidney disease, nephrolithiasis, malabsorption syndrome, hypertension, cancer, and nontraumatic fractures. With this matching strategy, we identified 16 156 matched nondonor individuals to whom surveys were sent.

### Surveys

We identified fracture outcomes through a survey sent to both the donors and the matched nondonors over a 2-year period between December 1, 2021, and July 31, 2023. Survey questions for fractures were derived from validated surveys used in the National Health and Nutrition Examination Survey, Framingham Study, Women’s Health Initiative, Study of Osteoporotic Fractures, and the Nurses’ Health Study.^33,34,35,36^ Several studies have demonstrated the ability of individuals to accurately recall fractures that occurred more than 10 years before the survey.^37,38,39,40^ In addition to fractures, the survey included questions on self-reported race and ethnicity, height, weight, smoking, alcohol use, menstrual status, self-reported osteoporosis diagnosis, and use of medications known to interfere with mineral metabolism, including glucocorticoids, antiepileptics, bisphosphonates, estrogen or progesterone, diuretics, rifampin, and vitamin D. Initial contact was made through mailed questionnaires with subsequent telephone follow-up. We used LexisNexis and Accurint^41^ to locate persons who were known to be alive but for whom no address could be found.

### Statistical Analysis

We report survey participation rates separately by health care site of kidney donation (for donors only) and by sex, age, and race and ethnicity (separately for donors and nondonor controls). For primary analysis of fractures, we compared donors and nondonors using a person-years approach to calculate the incidence rates of reported fractures from the date of donation (or index date for the nondonors) and the date of survey. We used fracture rates observed among nondonors to the donor group to calculate an expected number of fractures. To account for differences in the time under observation between donors and nondonors, fracture rates were calculated separately in strata defined by sex, age at time of donation or index, and years after index. Risk was assessed by calculating the corresponding standardized incidence ratio (SIR) and 95% CI.^42^ All *P* values were from 2-tailed tests, and results were deemed statistically significant at *P* < .05. All outcomes (types of fractures) were prespecified at the time of survey design, and as such, we do not directly adjust *P* values for multiple comparisons. However, readers are able to easily modify the threshold of significance when interpreting results.

To explore whether the nondonor controls who participated in the survey were representative of the full group of nondonor controls (ie, to assess volunteer bias), we identified a subset of 3773 controls who had 70% or more of the interval of time from index date through survey date covered by medical diagnostic information available as part of the REP. We then identified fractures reported in medical records during the same time interval using *International Classification of Diseases, Ninth Revision *and *International Statistical Classification of Diseases and Related Health Problems, Tenth Revision* diagnostic codes and code groupings defined by the Clinical Classifications Software to compare rates of fractures among those who participated in the survey vs those who did not.

Last, to assess the validity of the fracture survey instrument, we identified a subset of controls who completed surveys and who also had 70% or more of their time interval from index date to survey date covered by diagnostic information available as part of the REP. We identified a validation sample that included controls who reported a fracture on the survey (n = 166) and an equal number of controls who had reported no history of fractures on the survey (n = 166). A nurse abstractor reviewed the complete medical records of these 332 controls to assess the agreement of self-reported fractures on the survey with fractures identified in the medical records. Statistical analyses were performed using SAS, version 9.4 (SAS Institute Inc) and were completed from May to August 2023.

## Results

Surveys were sent to a total of 5065 donors and 16 156 matched nondonor controls (Table 1). A total 2132 donors (mean [SD] age, 67.1 [8.9] years; 1245 women [58.4%]) and 2014 controls (mean [SD] age, 68.6 [7.9] years; 1140 women [56.6%]) returned the surveys. Participation rates varied substantially for donors and controls (42.1% [2132 of 5065] vs 12.5% [2014 of 16 156]). Participation rates also varied by age of participants (rates were similarly highest among persons 67 to 72 years of age for both donors and controls) and were highest among White participants compared with persons of a racial or ethnic minority group or unknown race. Participation rates varied substantially across donation medical centers.

**Table 1.** Survey Participation Rates for Donors and Controls in Strata by Personal Characteristics

Characteristic	Donors			Nondonor controls		
	Surveys sent, No.	Surveys completed, No. (%)^a^^,^^b^	*P* value for difference^c^	Surveys sent, No.	Surveys completed, No. (%)^a^^,^^b^	*P* value for difference^c^
Overall	5065	2132 (42.1)	NA	16 156	2014 (12.5)	NA
Sex						
Female	2912	1245 (42.8)	.27	8946	1140 (12.7)	.23
Male	2153	887 (41.2)		7210	874 (12.1)	
Age, y^d^						
<61	1436	532 (37.0)	<.001	4237	406 (9.6)	<.001
61-66	1129	508 (45.0)		4059	507 (12.5)	
67-72	1051	533 (50.7)		3884	594 (15.3)	
≥73	1449	559 (38.6)		3976	507 (12.8)	
Race and ethnicity						
Racial or ethnic minority group	117	34 (29.0)	<.001	636	43 (6.8)	<.001
White	3594	1724 (48.0)		9245	1328 (14.4)	
Unknown	1354	374 (27.6)		6275	643 (10.3)	
Site of donation						
Mayo Clinic	2308	878 (38.0)	<.001	NA^e^	NA	NA
Hennepin	633	196 (31.0)		NA	NA	
University of Minnesota	2124	1058 (49.8)		NA	NA	

Abbreviation: NA, not applicable.

^a^ Survey completed and returned with a signed Health Insurance Portability and Accountability Act consent form.

^b^ Percentages are calculated across rows.

^c^ *P* value for differences in survey participation rates across characteristic categories (χ^2^ test of differences).

^d^ Age of person on January 1, 2022. Age was stratified by quartiles of integer age in the control group.

^e^ All matched controls were identified from the Rochester Epidemiology Project, regardless of the site of donation of the original donor.

**Figure 1.**
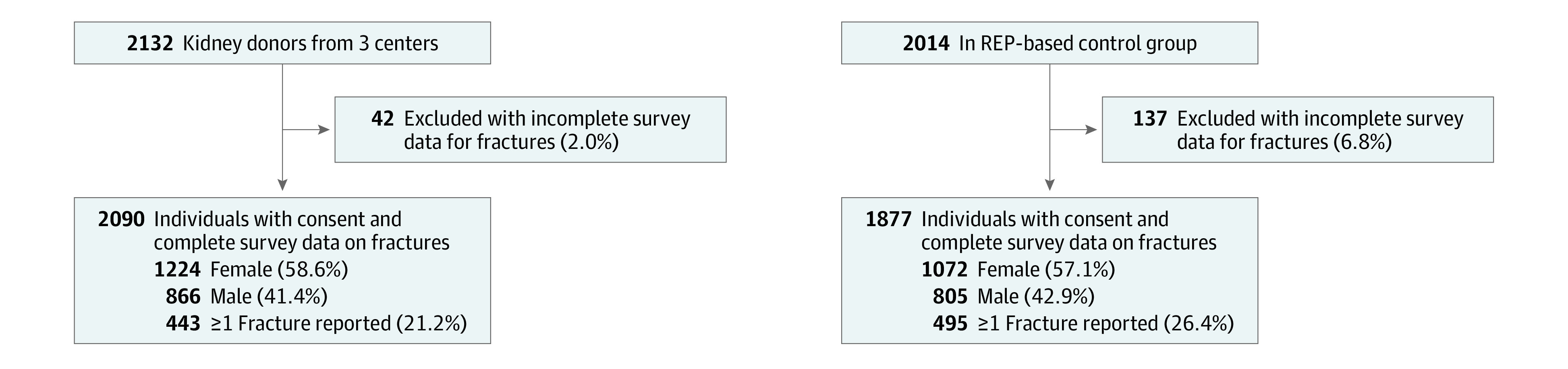
Inclusion of Donors and Controls in the Study Population REP indicates Rochester Epidemiology Project.

**Figure 2.**
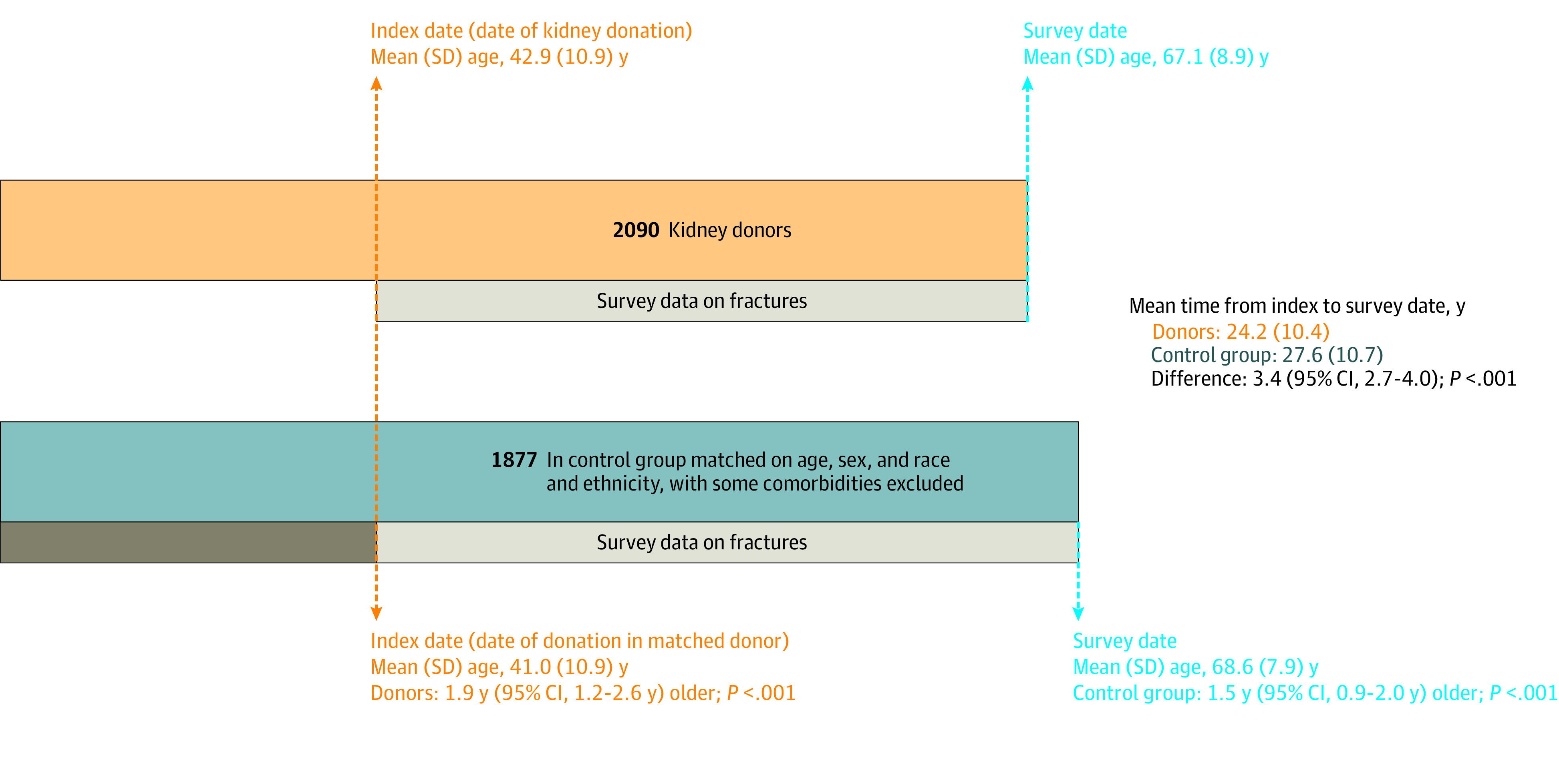
Schematic for Time Windows for Fractures Among Donors and Controls

In analyses of the risk of fractures, 42 donors and 137 controls were excluded from analyses because of incomplete survey data for fractures (Figure 1). Thus, all further analyses comparing the observed and expected number of fractures are limited to the 2090 donors and 1877 controls for whom complete survey information on fractures was available. For the 2090 donors and 1877 controls included in final analyses, the mean (SD) time between donation or index date and survey date was 24.2 (10.4) years for donors and 27.6 (10.7) years for controls, and controls were 1.5 years older than the donors (68.6 vs 67.1 years) (Figure 2). The rate of all types of fractures among the 2090 donors was significantly lower than among the 1877 controls, with 443 observed vs 499.8 expected fractures (SIR, 0.89; 95% CI, 0.81-0.97; *P* = .009). However, there were significantly more vertebral fractures among all donors than among controls, with 51 observed vs 36.0 expected vertebral fractures (SIR, 1.42; 95% CI, 1.05-1.83; *P* = .02) (Table 2). Among men, there were 21 observed vertebral fractures vs 12.5 expected (SIR, 1.67; 95% CI, 1.04-2.47; *P* = .04).

**Table 2.** Standardized Incidence Ratios (SIRs) for Fracture Risk Among Kidney Donors and Controls

Fracture site	Overall				Female				Male			
	Observed, No.^a^	Expected, No.^b^	SIR (95% CI)^c^	*P* value	Observed, No.^a^	Expected, No.^b^	SIR (95% CI)^c^	*P* value	Observed, No.^a^	Expected, No.^b^	SIR (95% CI)^c^	*P* value
Any fracture reported	443	499.8	0.89 (0.81-0.97)	.009	297	325.6	0.91 (0.81-1.02)	.10	146	174.2	0.84 (0.71-0.98)	.03
Fragility fracture	162	151.6	1.07 (0.91-1.24)	.42	111	103.6	1.07 (0.88-1.28)	.49	51	48.0	1.06 (0.79-1.37)	.70
Hip	22	21.2	1.04 (0.65-1.51)	.93	13	11.7	1.11 (0.59-1.80)	.78	9	9.5	0.95 (0.43-1.66)	.78
Lower arm or wrist	98	108.5	0.90 (0.73-1.09)	.29	74	78.7	0.94 (0.74-1.17)	.57	24	29.8	0.81 (0.52-1.16)	.25
Spine or back (vertebrae)	51	36.0	1.42 (1.05-1.83)	.02	30	23.5	1.28 (0.86-1.78)	.22	21	12.5	1.67 (1.04-2.47)	.04
Other fracture sites												
Upper leg (not hip)	15	12.5	1.20 (0.67-1.89)	.54	9	7.9	1.14 (0.52-2.01)	.78	6	4.6	1.31 (0.47-2.57)	.62
Knee (patella)	20	16.0	1.25 (0.76-1.86)	.37	17	13.2	1.29 (0.75-1.98)	.35	3	2.8	1.08 (0.20-2.64)	.99
Lower leg or ankle	89	110.5	0.81 (0.65-0.98)	.03	60	65.7	0.91 (0.70-1.16)	.45	29	44.8	0.65 (0.43-0.90)	.01
Foot (not toe)	84	80.9	1.04 (0.83-1.27)	.76	60	63.1	0.95 (0.73-1.21)	.67	24	17.8	1.35 (0.86-1.94)	.19
Tailbone	12	10.7	1.12 (0.58-1.85)	.77	9	8.3	1.09 (0.49-1.91)	.89	3	2.4	1.25 (0.23-3.05)	.87
Hand (not finger)	16	26.5	0.60 (0.34-0.94)	.02	12	20.1	0.60 (0.31-0.98)	.04	4	6.4	0.63 (0.16-1.39)	.24
Elbow	26	26.2	0.99 (0.65-1.41)	.92	23	15.5	1.49 (0.94-2.16)	.09	3	10.7	0.28 (0.05-0.69)	.003
Upper arm or shoulder	50	51.3	0.97 (0.72-1.26)	.82	34	35.3	0.96 (0.67-1.31)	.78	16	16.0	1.00 (0.57-1.55)	.99
Other	122	141.7	0.86 (0.71-1.02)	.09	71	80.3	0.88 (0.69-1.10)	.27	51	61.4	0.83 (0.62-1.07)	.16

^a^ Observed fractures among kidney donors.

^b^ Expected number of fractures among kidney donors by applying age, sex, and time after index-specific fracture rates observed among controls.

^c^ SIR for rate of fractures among kidney donors compared with rate among controls. A SIR of more than 1.0 means that more fractures were observed among kidney donors than among controls. A SIR of less than 1.0 means that fewer fractures were observed among kidney donors than among controls.

We performed 2 separate validation studies among the controls. First, we found that controls who returned surveys were similar to controls who did not return surveys with regard to the frequency and type of fractures diagnosed in their historical medical records (ie, assessment of volunteer bias; Table 3). Second, we validated the fracture survey instrument among a random sample of 332 controls who returned surveys (166 who reported any fracture and 166 who reported no fractures). There was good overall agreement of fractures reported on the survey vs fractures found by a nurse in the complete historical medical records (85.9% raw agreement), and the agreement was significantly better than would be expected by chance alone (κ = 0.72; 95% CI, 0.64-0.79).

**Table 3.** Fractures Diagnosed in the Electronic Medical Records of Controls Separated by Participation Status^a^

Type of fracture^c^	CCS category^c^	Fracture diagnosis, No. (%)^b^		Difference, % (95% CI)^e^
		Did not participate (n = 3101)^d^	Did participate (n = 672)^d^	
All types of fractures^f^	NA	870 (28.1)	191 (28.4)	−0.37 (−4.13 to 3.39)
Fracture of neck of femur (hip)	226	47 (1.5)	8 (1.2)	0.33 (−0.60 to 1.25)
Skull and face fractures	228	42 (1.4)	9 (1.3)	0.02 (−0.94 to 0.97)
Fracture of upper limb	229	411 (13.3)	93 (13.8)	−0.59 (−3.46 to 2.29)
Fracture of humerus	229-01	50 (1.6)	17 (2.5)	−0.92 (−2.18 to 0.35)
Fracture of radius and ulna	229-02	161 (5.2)	41 (6.1)	−0.91 (−2.88 to 1.06)
Other fracture of upper limb	229-03	364 (11.7)	83 (12.4)	−0.61 (−3.35 to 2.12)
Fracture of lower limb	230	383 (12.4)	86 (12.8)	−0.45 (−3.23 to 2.33)
Fracture of tibia and fibula	230-01	86 (2.8)	16 (2.4)	0.39 (−0.90 to 1.68)
Fracture of ankle	230-02	124 (4.0)	28 (4.2)	−0.17 (−1.83 to 1.49)
Other fracture of lower limb	230-03	317 (10.2)	73 (10.9)	−0.64 (−3.22 to 1.94)
Other fractures	231	414 (13.4)	83 (12.4)	1.00 (−1.76 to 3.76)
Fracture of vertebral column	231-01	246 (7.9)	51 (7.6)	0.34 (−1.87 to 2.56)
Fracture of ribs	231-02	98 (3.2)	11 (1.6)	1.52 (0.38 to 2.66)
Fracture of pelvis	231-03	29 (0.9)	5 (0.7)	0.19 (−0.54 to 0.92)
Other and unspecified fracture	231-04	165 (5.3)	39 (5.8)	−0.48 (−2.42 to 1.45)

Abbreviations: CCS, Clinical Classifications Software; NA, not applicable.

^a^ Fracture rates from diagnoses available in the electronic medical record were summarized for 3773 controls who had 70% or more of their person-time between index date and survey date covered by the Rochester Epidemiology Project. These 3773 controls were identified from among the 16 156 total controls to whom surveys were mailed. The purpose of this analysis was to evaluate whether controls with a history of fractures were more likely to participate than controls without a history of fractures (ie, volunteer bias).

^b^ Any diagnosis coded in the electronic medical record from the index date (ie, the date of donation in the matched donor) to the survey date.

^c^ Fractures were grouped using the *International Classification of Diseases, Ninth Revision* and *International Statistical Classification of Diseases and Related Health Problems, Tenth Revision* codes from the CCS categories.

^d^ Persons participated if they returned a completed survey and signed a Health Insurance Portability and Accountability Act consent form.

^e^ Percentage differences in types of fractures found in the medical records for controls who participated in the survey compared with those who did not participate.

^f^ Any fracture diagnosis from among the subtypes included in the lower part of the table.

## Discussion

Kidneys from living donors represent an important source of organs used for transplantation.^22^ Kidney donation is an altruistic act in which donors are prepared to accept small risks for the good of the recipient. Although it is well established that the long-term risk of chronic kidney disease is higher among donors vs nondonors, donors are prepared to accept these risks because they are relatively small.^3,4,5^ This survey-based study focused specifically on bone health among more than 2000 living kidney donors and suggests an excess risk of vertebral fractures after a mean follow-up of 25 years.

There are no studies with sufficiently long follow-up to date examining the risk of fractures among kidney donors, to our knowledge. A Canadian study did not demonstrate an excess risk of fractures among kidney donors, but the cohort included younger donors (mean age, 43 years) at low risk who had donated a mean of 6 years before participation in the study.^29^ Only 19% of the donors had a donor nephrectomy more than 12 years before study. We suggest that this study may have failed to observe an increased risk of fractures among donors because the excess risk of fractures would not be expected to occur until many years after donation and typically at older than 50 years of age. A prospective study measured markers of mineral and bone metabolism among 182 kidney donors and 173 paired controls after kidney donation.^20^ Donors had evidence of secondary hyperparathyroidism due to a reduction of serum 1,25(OH)_2_D with significantly higher serum intact parathyroid hormone and fibroblast growth factor 23 concentrations and significantly lower serum 1,25(OH)_2_D and inorganic phosphate concentrations, measured GFR, and reduced tubular reabsorption of phosphate 6 and 36 months after donation compared with healthy controls. Higher concentrations of bone resorption markers and the bone formation markers were observed among donors compared with healthy controls. A limited number of studies measuring hormonal and bone turnover variables have shown similar findings.^21,22,23,24,25,26^

The present study suggests a lower risk of all types of fractures but an excess risk of vertebral fractures among living kidney donors compared with controls after a mean follow-up of 25 years. Vertebral fractures would principally reflect deficits in trabecular bone, whereas all fractures, particularly in the axial skeleton, would principally reflect deficits in cortical bone.^43^ As such, our findings would point to possible deficits in trabecular bone among kidney donors, and direct studies addressing this possibility are warranted. The reduction in all types of fractures combined, which would be associated primarily with changes in cortical bone, could reflect either higher-quality cortical bone in the donors or perhaps altered activity patterns or other lifestyle changes after kidney donation. Mechanistically, it is difficult to explain increases in cortical bone among kidney donors, so altered activity or lifestyle patterns (eg, avoidance of higher-risk activities that may lead to long bone fractures) could be a potential explanation. Again, further studies are needed to evaluate these possibilities.

### Strengths and Limitations

This study has some strengths, including the large number of donors and matched controls and the long duration of follow-up after donation and index date. The metabolic abnormalities contributing to an increased fracture rate would have been present for a long time. Secondary hyperparathyroidism among donors is potentially treatable by therapy with vitamin D_3_ or activated vitamin D receptor agonists, such as calcitriol or paricalcitol. A trial of these medications among kidney donors may be necessary to determine whether they may offset the association of kidney donation with long-term bone health. Another strength of our study is the sampling frame available using the REP for selecting controls. This resource allowed us to restrict controls to those who did not have comorbidities that would have precluded kidney donation. The REP infrastructure allowed us to perform validation studies; we assessed the potential for volunteer bias among controls who returned surveys, and we assessed the validity of the fractures reported on the survey compared with fractures reported in medical records.

Our study also has some limitations. First, although we excluded nondonor controls based on medical history diagnoses, due to the historical nature of the study, we were not able to screen them for some other factors that would have precluded kidney donation, such as laboratory test and imaging results. In addition, despite knowing that the outcomes were all prespecified at the time of study design and survey design, some readers may wish to interpret the findings using a stricter threshold of statistical significance than α = .05 (ie, adjustments for multiple comparisons). We have provided all *P* values for such interpretation, and we recognize that our findings may be due to type 1 statistical errors (as is true for all statistical analyses). Validation studies were limited to controls because we do not have access to the complete historical medical records of the donors who currently reside across the US and receive health care elsewhere. The study population is limited to surviving donors and controls who were alive at the time of the survey. Hence, it is not feasible to use survival analyses methods. Inaccurate recall of the exact timing of fractures can be a potential concern, but it would not affect the results with the type of statistical analyses performed (ie, observed vs expected number of fractures irrespective of when the fractures occurred). Due to small numbers of racial or ethnic minority group participants, we were unable to perform stratified analyses by race and ethnicity. Finally, as a separate part of this study, we are also investigating the microstructure of bone among a subset of participants as further confirmation of our survey findings.

## Conclusion

In this survey-based study, we observed a reduction in overall fractures but an excess risk of vertebral fractures among living kidney donors compared with controls after a mean follow-up of 25 years. Treatment of excess vertebral fractures with dietary supplements such as vitamin D_3_ may reduce the numbers of vertebral fractures and patient morbidity.

## Notes

Supplement.
Data Sharing Statement
