# Supplementary material for: Fracture Risk Among Living Kidney Donors 25 Years After Donation
Source: JAMA Netw Open. 2024 Jan 24;7(1):e2353005. doi: 10.1001/jamanetworkopen.2023.53005 (PMC10809017; doi:10.1001/jamanetworkopen.2023.53005)
Supplement: Supplement. — Data Sharing Statement [file jamanetwopen-e2353005-s001.pdf]

Data Sharing Statement

Maradit Kremers. Fracture Risk Among Living Kidney Donors 25 Years After Donation. *JAMA Netw Open*. Published January 24, 2024. doi:10.1001/jamanetworkopen.2023.53005

Data
Data available: Yes
Data types: Deidentified participant data, Data dictionary
How to access data: Request for the data may be sent to the corresponding author, Dr. Rajiv Kumar - rkumar@mayo.edu
When available: With publication

Supporting Documents
Document types: None

Additional Information
Who can access the data: Researchers
Types of analyses: Any purpose
Mechanisms of data availability: With Investigator Support after approval and with a signed data access agreement.
